# Women’s Longitudinal Patterns of Smoking during the Pre-Conception, Pregnancy and Postnatal Period: Evidence from the UK Infant Feeding Survey

**DOI:** 10.1371/journal.pone.0153447

**Published:** 2016-04-25

**Authors:** Kate E. Fitzpatrick, Ron Gray, Maria A. Quigley

**Affiliations:** Policy Research Unit in Maternal Health and Care, National Perinatal Epidemiology Unit, Nuffield Department of Population Health, University of Oxford, Oxford, United Kingdom; National Cancer Center, JAPAN

## Abstract

**Background:**

An understanding of women’s longitudinal patterns of smoking during the pre-conception, pregnancy and postnatal period and the factors associated with these patterns could help better inform smoking cessation services and interventions.

**Methods:**

Latent class analysis (LCA) was used to empirically identify women’s smoking patterns in a sample of 10,768 mothers from the 2010 UK Infant Feeding Survey. Multinomial logistic regression was used to identify characteristics associated with these patterns.

**Results:**

LCA identified five distinct smoking patterns during the pre-conception, pregnancy and postnatal period: “non-smokers” (74.1% of women); “pregnancy-inspired quitters” (10.2%); “persistent smokers” (10.1%); “temporary quitters” (4.4%); and postnatal quitters (1.1%). Smoking patterns varied markedly according to socio-demographic variables and parity. After adjusting for these variables, mothers who lived during pregnancy with a partner who smoked were more likely to be temporary quitters (aOR 2.64, 95% CI 1.74–3.99) or persistent smokers (aOR 3.32, 95% CI 2.34–4.72) than pregnancy-inspired quitters. Mothers who lived during pregnancy with someone else other than a partner who smoked were more likely to be persistent smokers (aOR 2.34, 95% CI 1.38–3.97) or postnatal quitters (aOR 2.97, 95% CI 1.07–8.24) than pregnancy-inspired quitters. Mothers given information on how their partner could stop smoking if they lived during pregnancy with a smoking partner were less likely to be persistent smokers (aOR 0.42, 95% CI 0.27–0.65) than pregnancy-inspired quitters.

**Conclusion:**

Health professionals should ask about smoking at every opportunity, and refer women who self-report as current smokers to an evidence based smoking cessation service.

## Introduction

Smoking in pregnancy is associated with health risks for both the mother and infant, including increased risks of stillbirth, low birthweight and sudden infant death syndrome [1–6]. In England the prevalence of smoking at the time of delivery has steadily declined from 15.1% in 2006/07 to 12.0% in 2013/14 [7]. However, there is increasing evidence that a high proportion of women who quit smoking while pregnant relapse during the postpartum period, with a recent UK study suggesting a relapse rate of nearly 50% in the early postpartum period [8]. This has health consequences not only for the mother such as an increased risk of cancer [3], but has also been associated with a range of negative effects for the child including sudden infant death syndrome, asthma and a higher risk of the child becoming a smoker themselves [9].

A range of effective interventions are available to help pregnant women stop smoking [10, 11]. In the UK, such support is currently available to pregnant women through the free National Health Service (NHS) Stop Smoking Services, although only a small number of women take up the offer of help during pregnancy or after childbirth [12]. Furthermore, the relapse prevention interventions evaluated so far in women who have quit smoking spontaneously in early pregnancy have not been shown to be effective, although the use of incentives may offer a promising [13], although controversial [14], approach. An understanding of women’s longitudinal patterns of smoking during the pre-conception, pregnancy and postnatal period and the characteristics associated with these patterns could help better inform smoking cessation services and interventions. To date, however, this is an area where there has been limited investigation: Kahn et al [15] and Mumford et al [16] examined longitudinal smoking patterns during the pre-conception, perinatal and early parenting period in two large cohorts of women, nationally representative of US births; and to our knowledge, Munafò et al [17] is the only study to have examined longitudinal smoking patterns across the pre-conception, pregnancy and postnatal period in a large cohort of British women. However, Munafò et al only assessed a limited number of variables (four measures of psychosocial adversity) when examining characteristics associated with smoking patterns and the cohort comprised women who delivered in the early 1990s and as such may not reflect contemporary UK smoking patterns.

The aims of this study were therefore to identify women’s longitudinal patterns of smoking during the pre-conception, pregnancy and postnatal period and investigate what characteristics are associated with these patterns with a view to informing smoking cessation services and interventions.

## Methods

### Study sample

The study sample comprised mothers who participated in the 2010 UK Infant Feeding Survey (IFS) [18]. The IFS is a quinquennial survey primarily conducted to monitor trends in infant feeding practices in the UK. For the 2010 IFS, a sample of 30 760 births registered in the UK between August and October 2010 was selected. The IFS included all births registered in Wales and Northern Ireland and a random sample of those registered in England and Scotland during the specified period, with oversampling in England and Scotland of mothers from the lowest Index of Multiple Deprivation (IMD) quintile to try and ensure adequate representation of younger and lower socio-economic groups. The survey was administered by post and online in three stages between September 2010 and August 2011: Stage 1 was conducted when infants were approximately four to ten weeks old, Stage 2 when they were around four to six months old, and Stage 3 when they were around eight to ten months old. Mothers were only asked to complete the later stage of the survey if they had responded to the previous stage. The survey response rate was 51% at Stage 1, 80% at Stage 2, and 86% at Stage 3. Analysis was restricted to the sample of mothers who completed and returned all three stages of the survey (n = 10 768) although survey weights were used to take account of non-response (see Statistical Methods).

### Measures

At each stage of the survey, mothers were asked questions about their cigarette smoking behaviour: at stage 1, mothers who reported ever smoking cigarettes were asked to recall if they had smoked at all in the last two years, which roughly covers the period of their pregnancy and the year before conception. Women who responded “yes” to this question were asked “Do you smoke cigarettes at all now?” Those who responded “yes” were asked to recall if they smoked cigarettes at all during pregnancy, after they found out they were pregnant, whilst those who responded “no” were asked to recall when they finally gave up (ticking one of the following answers: before they knew they were pregnant; as soon as they found out they were pregnant; later on during the pregnancy; or after the birth). At stages 2 and 3, mothers were also asked the question “Do you smoke cigarettes at all now?” Information from these questions was used to identify mother’s smoking status (whether or not they smoked cigarettes at all) at six time points—one year before pregnancy, during pregnancy after confirmation of pregnancy, later in pregnancy and at stages 1–3 of the survey.

A range of socio-demographic and pregnancy-related variables was examined as potential variables that might be associated with smoking patterns based on the published literature [8, 19–24]. The socio-demographic variables investigated were ascertained by questions asked at stage 1 and included maternal age, marital status, ethnicity, age finished full-time education and socioeconomic status (defined by National Statistics Socio-Economic Classification (NS-SEC) using the woman’s current or most recent occupational information). The pregnancy-related variables examined were also ascertained by questions asked at stage 1 and included parity (number of viable pregnancies), the woman’s estimated weekly alcohol consumption during pregnancy, whether during pregnancy the women lived with a partner or other household member who smoked, whether during pregnancy the woman was given any information about smoking during pregnancy, and whether during pregnancy the woman was given information on how their partner could stop smoking if they lived with a partner who smoked.

### Statistical analysis

Latent class analysis (LCA) is a statistical method which posits that homogenous unobserved subgroups (latent classes) can be identified within a heterogeneous group using a set of observed (indicator) variables. Using information on women’s observed smoking status at six time points (see six indicator variables in Table 1), LCA was used to empirically identify and estimate the prevalence of women’s patterns of smoking during the pre-conception, pregnancy and postnatal period by identifying subgroups of women with similar smoking patterns. LCA estimates the response probability for each indicator variable (i.e. probability of smoking or not smoking at each of the six time points) according to latent class membership. It also estimates the proportion of individuals within a sample that are expected to belong to each latent class. Each individual is assigned a probability of being in each latent class and is then assigned to the class with the highest posterior probability (i.e. modal class) for subsequent analysis. Hence, LCA has the advantage of using smoking status at six time points to create the latent classes and allows for the uncertainty of each individual belonging to a latent class, [16, 17] rather than using arbitrary definitions of smoking patterns which would be based on the many possible combinations of smoking status at six time points.

**Table 1:** Characteristics of the study sample.

	Number (%^a^) of women (n = 10768)
SMOKING STATUS OF MOTHER	
**Smoking status 1 year before pregnancy**	
Non-smoker	8258 (73.5)
Smoker	2499 (26.5)
**Smoking status during pregnancy, after confirmation of pregnancy**	
Non-smoker	9522 (87.7)
Smoker	1050 (12.3)
**Smoking status later on during pregnancy**	
Non-smoker	9617 (88.7)
Smoker	955 (11.3)
**Smoking status at S1 survey, when baby around 4–10 weeks old**	
Non-smoker	9625 (87.2)
Smoker	1130 (12.8)
**Smoking status at S2 survey, when baby around 4–6 months old**	
Non-smoker	9547 (86.4)
Smoker	1221 (13.6)
**Smoking status at S3 survey, when baby around 8–10 months old**	
Non-smoker	9435 (85.3)
Smoker	1333 (14.7)
SOCIO-DEMOGRAPHIC CHARACTERISTICS OF MOTHER	
**Age (in years)**	
35 or older	2632 (19.4)
30–34	3783 (28.5)
25–29	2850 (28.0)
Under 25	1477 (24.1)
**Marital status**	
Married/civil partnership or cohabiting	9538 (85.7)
Single	1059 (13.4)
Widowed, divorced or separated	87 (0.9)
**Ethnic group**^**b**^	
White	9715 (86.6)
Non-White	815 (13.4)
**Age finished full-time education (in years)**	
Over 18	6318 (51.9)
17 or 18	2878 (30.1)
16 and under	1497 (18.0)
**NS-SEC (based on woman's occupation)**	
Managerial & professional	4696 (34.8)
Intermediate	2248 (19.6)
Routine & manual	2438 (26.7)
Never worked	567 (9.8)
Not classified	819 (9.1)
PREGNANCY-RELATED CHARACTERISTICS	
**Parity**	
One child	5307 (52.5)
Two or more children	5461 (47.5)
**Mother's estimated weekly alcohol consumption during pregnancy**	
Did not drink	6204 (63.5)
Drank less than one unit	3112 (29.6)
Drank one or more units	658 (6.9)
**During pregnancy mother lived with partner who smoked**	
No	8589 (77.5)
Yes	2179 (22.5)
**During pregnancy mother lived with someone else who smoked**	
No	10339 (93.4)
Yes	429 (6.6)
**During pregnancy mother given information about smoking during pregnancy**	
No	2703 (25.2)
Yes	7997 (74.8)
**Mother given information on how partner could stop smoking if lived with partner who smoked during pregnancy**	
No	1275 (12.3)
Yes	895 (10.2)
NA, did not live with partner who smoked	8589 (77.5)

^**a**^ weighted % of individuals with complete data.

^b^ women in Northern Ireland were not asked their ethnic group since 99% of mothers in the 2001 census were white, therefore they are all assumed to be white.

The number of latent classes was decided by fitting models with different numbers of latent classes and then considering model interpretability and model fit, parsimony and stability using the Akaike information criterion (AIC)[25], Schwarz Bayesian information criterion (BIC)[26], Consistent AIC (CAIC) [27], sample-size adjusted BIC (adjBIC) [28], entropy R^2^ [29], and G^2^ fit statistic (Table 2). Individuals do not need to have complete data on all indicator variables to be included in the latent class analysis, enabling maximum use of all the data. However, in the LCA, missing data are handled with a full-information likelihood technique that assumes data are missing at random. LCA models were therefore fitted first using all women (n = 10 768) and then using only the subset of women which had complete data on all indicator variables (n = 10 569). Both analyses yielded similar results and therefore only the results based on all women are reported.

**Table 2:** Model fit information for LCA models with 1–6 latent classes.

Number of latent classes	AIC	BIC	CAIC	adjBIC	Entropy R^2^	G^2^
1	29553.99	29597.7	29603.7	29578.63	1	29541.99
2	3077.222	3171.919	3184.919	3130.606	0.98402839	3051.222
3	1056.327	1202.014	1222.014	1138.456	0.97074606	1016.327
4	242.0042	438.6812	465.6812	352.8787	0.98395689	188.0042
5	**169.8091**	**417.4764**	**451.4764**	**309.4289**	**0.96975303**	**101.8091**
6	183.5062	482.1639	523.1639	351.8713	0.70815914	101.5062

Multinomial logistic regression was used to investigate what variables were associated with latent class membership (i.e. smoking pattern as a categorical outcome variable). When examining the association between the socio-demographic/pregnancy-related variables and latent class membership, the models included all women–smokers and non-smokers. This was a statistically efficient way of simultaneously comparing non-smokers, women who successfully quit smoking after becoming pregnant and those who followed other smoking trajectories. A full regression model was developed by including socio-demographic and pregnancy-related variables that have previously been associated with women’s smoking behavior. Variables remained in the full model if there was evidence (p<0.05) that they were associated with membership in at least one latent class; these variables are listed in Table 3. All analyses were carried out using Stata v13 software, with the LCA performed using the LCA Stata plugin[30]. All analyses took account of Stage 3 UK sample weights, which corrected for differential sampling by country, differential response rates to the stage 1 survey (with weights based on mother’s age and area deprivation status) and attrition at stage 2 and 3 (with weights based on variables that differed significantly between responders and non-responders at stages 2 and 3 compared with stage 1: feeding status, socio-economic classification, mother’s age, age of the baby, area deprivation quintiles, region, ethnicity, smoking and drinking behaviour and whether the baby was full term or premature).

**Table 3:** Association between socio-demographic and pregnancy-related variables and latent class membership.

	Temporary quitters during preg (Class 2) vs Pregnancy-inspired quitters	Non-smokers (class 3) vs Pregnancy-inspired quitters	Persistent smokers (class 4) vs Pregnancy-inspired quitters	Postnatal quitters (class 5) vs Pregnancy-inspired quitters
	aOR^a^ (95% CI)	aOR^a^ (95% CI)	aOR^a^ (95% CI)	aOR^a^ (95% CI)
SOCIO-DEMOGRAPHIC VARIABLES				
**Age of mother (in years)**				
35 or older	1	1	1	1
30–34	1.17 (0.70–1.98)	0.94 (0.73–1.22)	1.02 (0.66–1.58)	1.3 (0.51–3.30)
25–29	1.75 (1.03–2.98)*	0.76 (0.58–1.01)	1.31 (0.85–2.01)	1.38 (0.53–3.60)
Under 25	1.77 (0.93–3.35)	0.52 (0.37–0.74)***	1.32 (0.81–2.14)	0.75 (0.24–2.37)
**Marital status**				
Married/civil partnership or cohabiting	1	1	1	1
Single	1.68 (1.00–2.82)*	0.49 (0.35–0.68)***	1.89 (1.25–2.87)**	1.55 (0.59–4.07)
Widowed, divorced or separated	0.46 (0.12–1.84)	0.35 (0.14–0.88)*	1 (0.33–3.02)	0.67 (0.07–6.42)
**Ethnic group**^**b**^				
White	1	1	1	1
Non-White	0.69 (0.31–1.57)	2.42 (1.51–3.89)***	0.24 (0.09–0.60)**	0.75 (0.16–3.51)
**Age of mother when finished full-time education (in years)**				
Over 18	1	1	1	1
17 or 18	0.87 (0.58–1.31)	0.61 (0.48–0.77)***	1.37 (0.94–2.00)	1.03 (0.56–1.92)
16 and under	1.07 (0.67–1.71)	0.54 (0.40–0.71)***	2.74 (1.84–4.08)***	0.97 (0.42–2.28)
**NS-SEC (based on woman's occupation)**				
Managerial & professional	1	1	1	1
Intermediate	0.64 (0.39–1.04)	0.7 (0.54–0.90)**	0.89 (0.57–1.40)	0.62 (0.22–1.78)
Routine & manual	1.1 (0.70–1.71)	0.68 (0.52–0.89)**	1.91 (1.27–2.85)**	1.58 (0.58–4.34)
Never worked	1.73 (0.64–4.69)	1.84 (0.97–3.51)	7.58 (3.51–16.35)***	2.28 (0.43–12.16)
Not classified	0.9 (0.45–1.83)	0.76 (0.51–1.12)	1.59 (0.89–2.85)	2.26 (0.74–6.93)
PREGNANCY-RELATED VARIABLES				
**Parity**				
One child	1	1	1	1
Two or more children	1.97 (1.33–2.93)***	1.73 (1.40–2.13)***	2.98 (2.19–4.05)***	0.97 (0.48–1.93)
**Mother's estimated weekly alcohol consumption during pregnancy**				
Did not drink	1	1	1	1
Drank less than one unit	1.27 (0.85–1.88)	0.51 (0.41–0.64)***	1.28 (0.92–1.78)	1.46 (0.73–2.91)
Drank one or more units	1.23 (0.68–2.22)	0.43 (0.30–0.62)***	1.06 (0.60–1.88)	1.62 (0.64–4.11)
**During pregnancy lived with partner who smoked**				
No	1	1	1	1
Yes	2.64 (1.74–3.99)***	0.23 (0.18–0.30)***	3.32 (2.34–4.72)***	1.98 (0.96–4.10)
**During pregnancy lived with someone else who smoked**				
No	1	1	1	1
Yes	1.17 (0.55–2.46)	0.85 (0.51–1.41)	2.34 (1.38–3.97)**	2.97 (1.07–8.24)*
**During pregnancy, mother given information about smoking during pregnancy**				
No	1	1	1	1
Yes	0.91 (0.55–1.50)	0.53 (0.41–0.69)***	4.96 (2.94–8.36)***	1.14 (0.52–2.54)
**Mother given information on how partner could stop smoking if lived with partner who smoked during pregnancy**				
No	1	1	1	1
Yes	0.71 (0.42–1.21)	1.21 (0.84–1.73)	0.42 (0.27–0.65)***	0.67 (0.26–1.70)
NA, did not live with partner who smoked				

^a^ Adjusted for all variables in the table.

^b^ women in Northern Ireland were not asked their ethnic group since 99% of mothers in the 2001 census were white, therefore they are all assumed to be white.

* p<0.05

** p<0.01

*** p<0.001.

### Ethical approval

Our study was secondary data analysis of datasets from the IFS which are deposited in the UK Data Archive. The original survey was conducted by IFF Research and the University of York, on behalf of the government health departments of England, Northern Ireland, Scotland and Wales. Ethical approval to conduct the original survey was granted by the Ethics Committee, Department of Health Sciences at the University of York.

## Results

### Characteristics of study sample

Table 1 shows the observed smoking status of mothers at various time points before, during and after pregnancy, together with further sample description. Of the 10 768 mothers who responded to all three stages of the survey, 26.5% smoked one year prior to pregnancy, with the proportion dropping after confirmation of pregnancy to 12.3% and dropping slightly more during pregnancy to 11.3% and gradually increasing with time postnatally to 14.7% at the time of the third stage of the IFS (around 8–10 months postnatally). However, these proportions are measured separately at each time point and do not take account of changing smoking status over time in individual women. Nearly a quarter of women (22.5%) lived during pregnancy with a partner who smoked; 6.6% of all women (39% of women aged under 20) lived with someone else who smoked.

### Women’s longitudinal patterns of smoking

**Fig 1:**
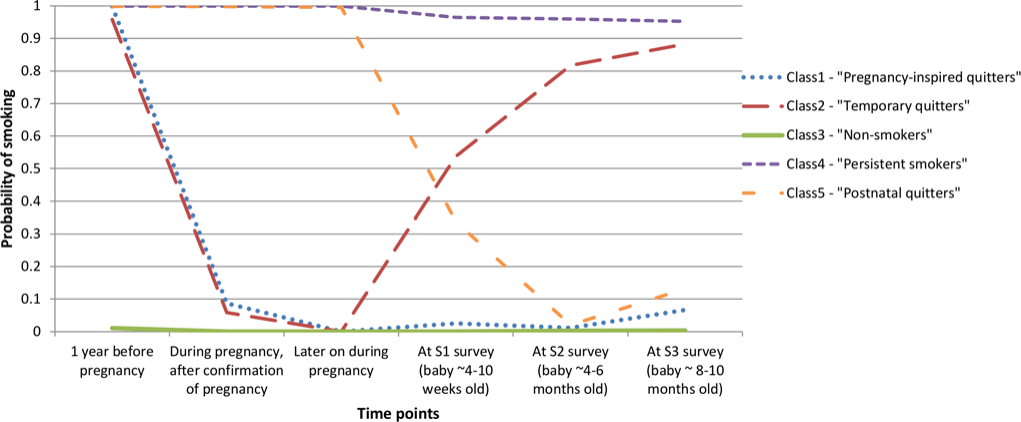
Probability of smoking at particular time points before, during and after pregnancy according to latent class membership.

Using the information on women’s observed smoking status outlined in Table 1, latent class models were fitted to the data starting with the most parsimonious one-class model (all women show the same pattern of smoking) and progressing to less parsimonious models with an increasing number of classes (i.e. number of distinct patterns of smoking). Having considered the model fit information (Table 2) and model interpretability, the five-class model was selected. The estimated probability of smoking at particular time points before, during and after pregnancy given class membership is shown in Fig 1. An estimated 10.2% (n = 1100) of mothers belonged to class 1, characterised by a high probability of smoking prior to pregnancy and a low probability of smoking during and after pregnancy (i.e. not relapsing postnatally), subsequently referred to as the “pregnancy-inspired quitters”. An estimated 4.4% (n = 479) of mothers belonged to class 2, characterised by a high probability of smoking prior to pregnancy, a low probability of smoking during pregnancy and an increasingly high probability of smoking with time postnatally (i.e. relapsing postnatally), subsequently referred to as the “temporary quitters”. Nearly three-quarters of mothers (74.1%, n = 7981) were estimated to belong to class 3, characterised by a low probability of smoking at each time point, subsequently referred to as the “non-smokers”. The 10.1% (n = 1087) of mothers estimated to belong to class 4, had a high probability of smoking at each time point and are subsequently referred to as the “persistent smokers”. Finally, an estimated 1.1% (n = 122) of mothers belonged to class 5, distinguished by a high probability of smoking prior to and during pregnancy but a low probability of smoking postnatally, subsequently referred to as the “postnatal quitters”.

Hence, an estimated 11.2% of mothers smoked during pregnancy (adding classes 4 and 5), and of the 1,579 mothers who quit during pregnancy (classes 1 and 2), 479 women (30.3%) relapsed by 8–10 months postnatally.

### Variables associated with women’s longitudinal patterns of smoking

From a smoking intervention point of view it is of particular interest to know what distinguishes between women who successfully quit smoking after becoming pregnant and those who follow other smoking trajectories. Pregnancy-inspired quitters (n = 1,100) were therefore used as the reference group when examining the association between selected variables and latent class membership (i.e. smoking pattern) (Table 3; numbers and percentages shown in S1 Table).

In unadjusted analysis (data not shown), all socio-demographic and pregnancy-related variables were significantly associated with membership in at least one of the latent classes. For example, about half of women who were persistent smokers, temporary quitters or postnatal quitters lived with a partner who smoked, compared with 37.8% of pregnancy-inspired quitters and 13.5% of non-smokers (S1 Table). Similarly, 22.1% of persistent smokers and 17.9% of postnatal quitters lived with someone else who smoked compared with 8.8% of pregnancy-inspired quitters (S1 Table). In adjusted analysis, all socio-demographic and pregnancy-related variables were independently associated with membership in at least one of the latent classes, although the effects were attenuated or strengthened in some instances by adjustment.

Compared to pregnancy-inspired quitters, persistent smokers were more likely to be single, have white ethnicity, have finished full-time education aged 16 or under, be in routine or manual occupations or never worked, and be parous (Table 3). After adjustment for these variables, they were more likely to have lived during pregnancy with a partner (aOR 3.32, 95% CI 2.34–4.72) or someone else who smoked (aOR 2.34, 95% CI 1.38–3.97) and be given information about smoking during pregnancy (aOR 4.96, 95% CI 2.94–8.36) whilst being less likely to be given information on how their partner could stop smoking if they lived with a smoking partner (aOR 0.42, 95% CI 0.27–0.65). Temporary quitters were more likely than pregnancy-inspired quitters to be 25-29yrs old, single, and parous. After adjustment for these variables, they were also more likely to be living with a partner who smoked (aOR 2.64, 95% CI 1.74–3.99). Postnatal quitters were only distinguishable from pregnancy-inspired quitters by being more likely to have lived with someone else who smoked (aOR 2.97, 95% CI 1.07–8.24).

By contrast, non-smokers were less likely than pregnancy-inspired quitters to be aged under 25yrs, single, widowed, divorced or separated (rather than married or co-habiting), have white ethnicity, have finished full-time education at a young age, be in intermediate or routine and manual occupations, be non-parous, and to have drank alcohol during pregnancy. After adjustment for these variables, they were less likely to have lived with a partner who smoked (aOR 0.23, 95% CI 0.18–0.30) and to have been given info about smoking during pregnancy (aOR 0.53, 95% CI 0.41–0.69).

## Discussion

### Summary of findings

Our study suggests that women exhibit one of five distinct patterns of smoking during the pre-conception, pregnancy and postnatal period: most prevalent (74.1%) were the “non-smokers”; an estimated 10.2% were “pregnancy-inspired quitters” with a tendency not to relapse postnatally; 10.1% were estimated to be “persistent smokers”; 4.4% were estimated to be “temporary quitters” with a tendency to relapse postnatally; and 1.1% were estimated to be “postnatal quitters”. Smoking patterns varied significantly according to socio-demographic variables and parity. After adjustment for these variables, we found that mothers who lived during pregnancy with a partner who smoked were more likely to be temporary quitters or persistent smokers than pregnancy-inspired quitters. Mothers who lived during pregnancy with someone else other than a partner who smoked were also more likely to be persistent smokers or postnatal quitters than pregnancy-inspired quitters. In addition, compared to pregnancy-inspired quitters, persistent smokers were less likely to have been given information on how their partner could stop smoking if they lived during pregnancy with a smoking partner.

### Comparison with other studies

Mumford et al [16] also identified five distinct patterns of smoking when they modeled smoking status from pre-conception until the child started formal schooling in a sample of US mothers. One of the patterns Mumford et al identified was referred to as “delayed initiators”, characterised by a low probability of smoking pre-conception, during pregnancy and at 9 months postpartum but an increasingly high probability of smoking as the child aged. We did not detect this pattern using smoking status data up to 8–10 months postpartum. The other four patterns identified by Mumford et al correspond with our “non-smoker”, “pregnancy-inspired quitter”, “persistent smoker” and “temporary quitter” patterns, but Mumford et al did not detect our identified pattern of “postnatal quitters”. When Munafò et al [17] modeled smoking status from pre-conception to 33 months postpartum in a sample of British mothers, however, they did identify a pattern equivalent to our “postnatal quitters” in addition to six other smoking patterns. Three of these other smoking patterns correspond to our “non-smoker”, pregnancy-inspired quitter” and “persistent smoker” patterns, although Munafò et al also identified three patterns of “temporary quitter” distinguished by when they stopped and when they relapsed to smoking in contrast to our one pattern of “temporary quitting”. This disparity may reflect differences between the studies in terms of the number and timing of observed smoking status data points examined.

The prevalence of smoking in pregnancy observed in our study is consistent with other UK data from the same period. For example, the proportion of mothers smoking later on during pregnancy in our study (11%, Table 1) was roughly comparable to national data for England on the proportion of women smoking at delivery in 2010/2011 (13.5%) [31]. There have been few UK studies of women’s longitudinal patterns of smoking during pregnancy and the postnatal period. In a study of 6,437 pregnant women in 2008–2009 in Southeast England [8], three-quarters of the women (74%) were non-smokers (consistent with the 74% prevalence of non-smokers observed in our study). The same study observed 8% of women quitting at any stage of pregnancy and remaining abstinent for the duration (similar to the 10% prevalence of pregnancy-inspired quitters in our study) and 46% of these women (3.7% of all women) relapsing by 6 weeks postnatally (similar to the 4.4% prevalence of temporary quitters in our study).

Another large UK cohort of mothers who gave birth in 2000–2001 (22) observed a higher prevalence of relapse at 9 months postnatally (57%) than our estimate of 30% at 8–10 months postnatally. Similarly, US data from 10 states in the 1990s (23) suggests that half of women relapse by 6 months postnatally. The British study which used LCA to identify smoking patterns [17] observed different prevalences than our study because smoking in pregnancy was more common in their study period (the early 1990s) than in ours (2010). For example, they observed a higher prevalence of smoking in pregnancy (approximately 19%, compared with 11.3% in our study) and a slightly higher prevalence of women who gave up smoking during pregnancy, but had relapsed by 8 months postnatally (approximately 6%, compared with the 4.4% temporary quitters in our study). They did, however, observe a similar prevalence of “postnatal quitters” (1.3%) to that observed in our study (1.1%).

The characteristics our study found to be associated with women’s smoking patterns are consistent with previous reviews which suggest that predictors of smoking among pregnant women include younger maternal age [20, 21], single marital status [20], lower level of education [19–21], lower socio-economic status [19–21], higher parity [19–21] and having a partner who smokes [19–21]. The more limited number of studies that have investigated predictors of smoking relapse in the postpartum period, suggest that many of the same characteristics, including being single, parous or having a partner who smokes, are also associated with smoking relapse in the postpartum period [8, 22–24, 32], consistent with our findings.

A recent synthesis of 38 qualitative studies [33] highlighted the importance of partners, together with family and friends, as a potential barrier or facilitator for quitting during pregnancy and sustained quitting postpartum. The synthesis also highlighted the mother’s changing connections with the baby as an important influence. For example, many women were motivated to quit smoking during pregnancy to protect the baby, and some sustained this postpartum in order to be ‘good mothers’ (perhaps reflected in our ‘pregnancy-inspired quitters’) whereas for others, quitting smoking during pregnancy was seen as something of a temporary change, undertaken for the baby during pregnancy (perhaps reflected in our ‘temporary quitters’).

### Strengths and limitations

Key strengths of our study are that our findings are based on a large, contemporary, national cohort of mothers. We had a large enough sample size (n = 10,768) to identify five distinct smoking patterns and identify characteristics associated with them. Although the initial response rate was 51%, with some attrition at later stages, we used survey weights, which were based on a large number of variables, to take account of differential sampling, differential response rates among different groups and non-response bias introduced through attrition over the course of the survey. A potential limitation is that mother’s smoking status was based on self-report, which could have led to under-reporting given the social stigma of smoking in pregnancy [34] and this may have affected the prevalence estimates for the different smoking patterns. The gold standard for measuring smoking status is cotinine, but this was not available in our study. Furthermore, mother’s smoking status at time points before and during pregnancy were retrospectively recalled at 4–10 weeks postpartum, introducing the possibility that women may not have accurately recalled this information. However, as discussed earlier, the prevalence of smoking patterns was similar to national data [31] and another large study in England [8]. Another limitation is that we were unable to examine variations in the longitudinal patterns of the amount smoked, only whether or not women smoked at all. Although we had data on a wide range of variables potentially associated with women’s smoking patterns, there were some potentially important variables (e.g. degree of tobacco addiction [19], changes in smoking status of the partners during pregnancy, or details of the information that the mothers received) that we did not have data on. Finally, as all of the data, including partner’s smoking status, were collected postnatally by maternal self-report, it is not possible to infer causality between mothers receiving information about smoking cessation and her smoking behaviour or that of her partner’s.

## Conclusions

Some women appear to exhibit marked fluctuations in smoking during the pre-conception, pregnancy and postnatal period suggesting the need for health professionals to ask about smoking at every opportunity, and refer women who self-report as current smokers to an evidence based smoking cessation service. Women at high risk of relapsing postnatally, such as those who are single or parous, can be identified antenatally as needing more support in the postnatal period. Our findings also suggest that pregnant women may be more likely to successfully quit smoking if partners and other household members who smoke are supported to stop smoking. Nearly a quarter of women (22.5%) lived during pregnancy with a partner who smoked; 6.6% of all women (39% of women aged under 20) lived with another household member who smoked). NICE [12] currently recommends that health professionals ask women if anyone else in their household smokes. If her partner or others in the household smoke, health professionals are advised to suggest they contact NHS Stop Smoking Services. However, a recent systematic review [35] found few effective interventions for encouraging partners who smoke to stop smoking and none of the identified studies included household members other than the expecting father, suggesting the need for effective interventions in this area. Finally, our findings suggest that measuring smoking at a single point in time during the pre-conception, pregnancy and postnatal period may not be a very accurate way of evaluating smoking interventions or the effects of smoking on the child given the complex nature of the smoking patterns.

## Supporting Information

S1 Table Socio-demographic and pregnancy-related characteristics of latent classes. (DOCX)
