# Supplementary material for: Women’s Longitudinal Patterns of Smoking during the Pre-Conception, Pregnancy and Postnatal Period: Evidence from the UK Infant Feeding Survey
Source: PLoS One. 2016 Apr 25;11(4):e0153447. doi: 10.1371/journal.pone.0153447 (PMC4844127; doi:10.1371/journal.pone.0153447)
Supplement: S1 Table — (DOCX) [file pone.0153447.s001.docx]

**Supporting Information**

**S1 Table. Socio-demographic and pregnancy-related characteristics of latent classes**

|  | n (%**^a^**) of Pregnancy inspired-quitters (class1) | n (%**^a^**) of Temporary quitters during pregnancy (class2) | n (%**^a^**) of Non-smokers (class3) | n (%**^a^**) of Persistent smokers (class4) | n (%**^a^**) of Postnatal quitters (class5) |
| --- | --- | --- | --- | --- | --- |
| SOCIO-DEMOGRAPHIC CHARACTERISTICS OF MOTHER |  |  |  |  |  |
| **Age of mother (in years)** |  |  |  |  |  |
| 35 or older | 202 (13.9) | 60 (10.1) | 2221 (22.2) | 134 (10.1) | 15 (9.3) |
| 30-34 | 345 (22.7) | 108 (17.8) | 3127 (32.2) | 184 (13) | 19 (18.4) |
| 25-29 | 340 (29.1) | 131 (31.1) | 2115 (28.1) | 242 (24.3) | 22 (33.4) |
| Under 25 | 261 (34.3) | 110 (41) | 767 (17.5) | 313 (52.6) | 26 (38.9) |
| **Marital status** |  |  |  |  |  |
| Married/civil partnership or cohabiting | 963 (80.9) | 318 (74.9) | 7597 (90) | 593 (65.1) | 67 (76.6) |
| Single | 164 (17.8) | 81 (24) | 541 (9.2) | 259 (33.5) | 14 (22.7) |
| Widowed, divorced or separated | 13 (1.3) | 8 (1.2) | 51 (0.8) | 14 (1.4) | 1 (0.8) |
| **Ethnic group (assuming northern Ireland all white)** |  |  |  |  |  |
| White | 1099 (94.7) | 389 (95.5) | 7302 (83.1) | 848 (97.9) | 77 (95.9) |
| Non-White | 35 (5.3) | 16 (4.5) | 748 (16.9) | 13 (2.1) | 3 (4.1) |
| **Age of mother when finished full-time education (in years)** |  |  |  |  |  |
| Over 18 | 527 (39.7) | 172 (34.6) | 5377 (59.8) | 207 (17.8) | 35 (36.6) |
| 17 or 18 | 396 (40.1) | 138 (39) | 1968 (26.8) | 343 (37.6) | 33 (42.3) |
| 16 and under | 215 (20.2) | 99 (26.5) | 847 (13.4) | 322 (44.6) | 14 (21.1) |
| **NS-SEC (based on woman's occupation)** |  |  |  |  |  |
| Managerial & professional | 382 (27.3) | 120 (21.8) | 4031 (40.2) | 139 (10.6) | 24 (18.7) |
| Intermediate | 276 (24.3) | 90 (18.5) | 1702 (19.8) | 167 (13.6) | 13 (14.3) |
| Routine & manual | 362 (34.4) | 146 (42.4) | 1519 (21.9) | 383 (45.2) | 28 (41.3) |
| Never worked | 33 (3.8) | 23 (7.8) | 391 (9.4) | 115 (20.5) | 5 (8.8) |
| Not classified | 97 (10.3) | 30 (9.6) | 608 (8.7) | 72 (10.1) | 12 (16.9) |
| PREGNANCY-RELATED CHARACTERISTICS |  |  |  |  |  |
| **Parity** |  |  |  |  |  |
| One child | 736 (68.2) | 225 (55.2) | 3892 (49.9) | 403 (51.3) | 51 (68.1) |
| Two or more children | 414 (31.8) | 184 (44.9) | 4359 (50.1) | 473 (48.7) | 31 (31.9) |
| **Mother's estimated weekly alcohol consumption during pregnancy** |  |  |  |  |  |
| Did not drink | 575 (54.3) | 196 (53) | 4896 (66.7) | 503 (56.6) | 34 (48.1) |
| Drank less than one unit | 386 (35.9) | 142 (37.9) | 2314 (27.3) | 239 (35.5) | 31 (40.8) |
| Drank one or more units | 100 (9.8) | 32 (9.1) | 437 (6.1) | 79 (8) | 10 (11.2) |
| **During pregnancy lived with partner who smoked** |  |  |  |  |  |
| No | 729 (62.2) | 190 (43.7) | 7240 (86.5) | 389 (45.6) | 41 (49.5) |
| Yes | 421 (37.8) | 219 (56.3) | 1011 (13.5) | 487 (54.4) | 41 (50.5) |
| **During pregnancy lived with someone else who smoked** |  |  |  |  |  |
| No | 1080 (91.2) | 375 (90.2) | 8057 (96.2) | 754 (77.9) | 73 (82.1) |
| Yes | 70 (8.8) | 34 (9.8) | 194 (3.8) | 122 (22.1) | 9 (17.9) |
| **During pregnancy, mother given information about smoking during pregnancy** |  |  |  |  |  |
| No | 184 (16.2) | 67 (18.5) | 2390 (29.9) | 50 (4.8) | 12 (18.4) |
| Yes | 961 (83.8) | 341 (81.5) | 5799 (70.1) | 826 (95.2) | 70 (81.6) |
| **Mother given information on how partner could stop smoking if lived with partner who smoked during pregnancy** |  |  |  |  |  |
| No | 229 (17.9) | 143 (31.5) | 576 (7.1) | 300 (33.4) | 27 (32.7) |
| Yes | 190 (19.8) | 76 (24.8) | 428 (6.3) | 187 (21) | 14 (17.9) |
| NA, did not live with partner who smoked | 729 (62.3) | 190 (43.7) | 7240 (86.6) | 389 (45.6) | 41 (49.5) |
| **^a^**weighted % of individuals with complete data | |  |  |  |  |
